# Biosecurity practices on Australian commercial layer and meat chicken farms: Performance and perceptions of farmers

**DOI:** 10.1371/journal.pone.0195582

**Published:** 2018-04-18

**Authors:** Angela Bullanday Scott, Mini Singh, Peter Groves, Marta Hernandez-Jover, Belinda Barnes, Kathryn Glass, Barbara Moloney, Amanda Black, Jenny-Ann Toribio

**Affiliations:** 1 University of Sydney, Camden, Australia; 2 Charles Sturt University, Wagga Wagga, Australia; 3 Department of Agriculture, Canberra, Australia; 4 Australian National University, Canberra, Australia; 5 New South Wales Department of Primary Industries, Sydney, Australia; University of Campinas, BRAZIL

## Abstract

This paper describes the level of adoption of biosecurity practices performed on Australian commercial chicken meat and layer farms and farmer-perceived importance of these practices. On-farm interviews were conducted on 25 free range layer farms, nine cage layer farms, nine barn layer farms, six free range meat chicken farms and 15 barn meat chicken farms in the Sydney basin bioregion and South East Queensland. There was a high level of treatment of drinking water across all farm types; town water was the most common source. In general, meat chicken farms had a higher level of adoption of biosecurity practices than layer farms. Cage layer farms had the shortest median distance between sheds (7.75m) and between sheds and waterbodies (30m). Equipment sharing between sheds was performed on 43% of free range meat chicken farms compared to 92% of free range layer farms. There was little disinfection of this shared equipment across all farm types. Footbaths and visitor recording books were used by the majority of farms for all farm types except cage layer farms (25%). Wild birds in sheds were most commonly reported in free range meat chicken farms (73%). Dogs and cats were kept across all farm types, from 56% of barn layer farms to 89% of cage layer farms, and they had access to the sheds in the majority (67%) of cage layer farms and on the range in some free range layer farms (44%). Most biosecurity practices were rated on average as ‘very important’ by farmers. A logistic regression analysis revealed that for most biosecurity practices, performing a practice was significantly associated with higher perceived farmer importance of that biosecurity practice. These findings help identify farm types and certain biosecurity practices with low adoption levels. This information can aid decision-making on efforts used to improve adoption levels.

## Introduction

Biosecurity refers to actions and measures implemented to prevent and control the introduction and spread of infectious diseases. Good biosecurity is vital to the successful performance of any poultry production system [1]. Biosecurity protocols can apply to several sections of a poultry production system including: personnel and visitor requirements, restricting contact between poultry and other animals, proper shed sanitisation, equipment and vehicle disinfection, and thorough water treatment. Biosecurity protocols on poultry production systems aim firstly to reduce the potential introduction of infectious diseases on the farm. If an infectious disease is introduced and becomes established, the protocols aim to limit spread of the disease within and between farms. Proper implementation of biosecurity protocols maintains good health and welfare of poultry on farms and reduces financial losses by decreasing the frequency and magnitude of infectious disease outbreaks [2–4].

The Australian poultry industry has experienced several outbreaks of emergency animal diseases (EADs); namely highly pathogenic avian influenza (HPAI) and Newcastle disease (ND) [5]. EADs have the potential to severely impact Australia’s economy and close the trade and markets of animal products. Australia has had seven HPAI outbreaks recorded since 1976; the latest occurred in the state of New South Wales (NSW) in 2013 which alone cost up to $4 million to eradicate [6, 7]. The Emergency Animal Disease Response Agreement (EADRA) was developed as a contractual arrangement between Australian commonwealth, state and territory governments and livestock industry groups to prepare for and respond to EADs. The EADRA includes a commitment from all groups to detect and alert suspect EADs, participate in EAD responses and minimise the occurrence of an EAD, particularly through the development and implementation of biosecurity protocols and plans. Biosecurity manuals are a component of this preparedness and are under regular revision [8]. A national farm biosecurity manual exists for the Australian poultry industry which stipulates minimum biosecurity standards that must be applied on all Australian poultry farms [1]. Additional biosecurity manuals exist for the different sectors of the Australian poultry industry to highlight specific and additional biosecurity standards for farms with different species (e.g. ducks and turkeys) and different production types (e.g. meat and eggs) [4, 9]. Adoption and compliance with these standards occurs at the farm level. The adoption levels of biosecurity practices in the Australian poultry industry were previously assessed via phone and in-person surveys of farms in 2005. The adoption levels were regarded as high in the chicken meat sector but no assessment was made on the chicken egg sector due to a low response rate within this sector. The lower response rate was most likely due to the high proportion of private ownership in the egg sector compared to the chicken meat sector, and this greater diversity in ownership likely creates greater variation in management practices and farm design across the layer sector. In comparison, the Australian meat chicken industry is vertically integrated, and 70% of Australian’s chicken meat production is owned by two companies [4, 10].

In recent years there has been a significant consumer-driven expansion of free range poultry production in Australia. Other developed countries have also experienced an increase in free range poultry production, particularly countries of the European Union and the United Kingdom where a ban on battery cages has been implemented since 2012. Farms in these countries must house chickens in enriched cages or use alternative systems such as barn or free range [11]. Australian free range meat chicken production, regarded as a ‘cottage industry’ in 2006, now accounts for at least 15% of the total market [12]. Similarly, the retail market share of free range chicken eggs has increased from 10% in 2000 to 40% in 2015 [13]. There is concern amongst poultry veterinarians and industry experts that this growing trend will increase the occurrence of diseases in the poultry industry. Wild birds are known to harbor a number of bacterial, fungal, viral and parasitic diseases that can be transmitted to poultry, and free-range production may increase the potential for diseases to transfer from wildlife to poultry when poultry will have access to the outdoors [10, 14]. Of particular concern to the Australian poultry industry is the avian influenza (AI) virus [7, 10, 15].

AI is currently circulating in Australian wild birds as low pathogenic AI (LPAI) at an approximate 2% prevalence overall; highest in waterfowl species such as ducks and geese at an approximate 2.5% prevalence. In contrast to waterfowl in the northern hemisphere which are known to perform annual migrations over long distances, the movements of Australian waterfowl species are less predictable and most populations are nomadic where movements are confined to Australia and occur in response to rainfall events. All Australian HPAI outbreaks thus far were caused by the introduction and subsequent mutation of unique Australian lineages of LPAI virus; no exotic lineages have been detected in Australia [16, 17]. Globally, most historical HPAI outbreaks have had little geographical spread. There are major exceptions to this, such as the HPAI H5N1 epizootic which started in 2003, extended to over 60 countries and remains endemic in Bangladesh, China, Egypt, Indonesia, Vietnam and large parts of eastern India. The mechanisms of spread are largely due to human movement of poultry and poultry products, and the role of wild birds in the introduction and spread of HPAI H5N1 is controversial [18]. Other major exceptions are the HPAI outbreaks that occurred in the USA in 2015. HPAI H5N8 was first detected in a flock of mixed poultry in Oregon in December 2014. This virus was of Eurasian origin, most likely introduced from intercontinental wild bird migrations and spread rapidly along wild bird migratory pathways. There was mixing of the H5N8 with North American strains which generated new HPAI combinations, such as the HPAI H5N2 detected afterwards [19, 20]. HPAI H5N2 was first detected in a backyard flock of mixed poultry in Washington State in January 2015. Outbreaks of both viruses affected many poultry farms in more than 15 states of the USA and continued until mid-2016.

A research project was commenced in 2015 to quantify the likelihood of AI virus introduction and spread between different types of commercial chicken farms in Australia and to evaluate the reduction in likelihood of alternate on-farm mitigation actions. An on-farm survey was conducted involving interviews and observations during farm visits to the main types of Australian commercial chicken farms: barn meat chicken, free range meat chicken, cage layer, barn layer and free range layer farms. The objective of the on-farm survey was to describe farm design, structure, wild animal exposure, contact with other farms and biosecurity practices among the farm types to inform understanding of variation between these farms that could impact on the risk of AI introduction and spread. This paper describes the data collection activities undertaken for this project and reports the results of the biosecurity components of the on-farm survey. These biosecurity components are relevant to limiting the introduction and spread of all infectious poultry diseases that can occur in Australian commercial chicken farms.

## Methods

### Farm survey

A survey which included on-farm interviews was conducted on commercial chicken farms in the Sydney basin and South-East Queensland regions of Australia from June 2015 to February 2016. In total, 25 free range layer farms, nine cage layer farms, nine barn layer farms, 15 free range meat chicken farms and 15 barn meat chicken farms were visited. Commercial layer farms were defined as those having more than 1,000 birds, and commercial meat chicken farms were defined as those having more than 25,000 birds. Details on the general methodology of the survey, including the region and farm selection, questionnaire development and conduct of the on-farm interviews can be found in Scott et al. (2017).

In brief, two questionnaires were created: a main questionnaire and a biosecurity questionnaire, both written in English and conducted on-farm via face-to-face interviews by the researchers. Interviewees were either farm owners or farm managers. The main questionnaire consisted of sections which captured data related to general farm information, water source and use, poultry health, range information, and farmer observations of wild birds and other wild animals. Distances were measured using Google Maps (2016 Google Inc., California, USA) after the farm visit.

The biosecurity questionnaire consisted of sections which captured data on biosecurity practices performed on the farm, farmer perceptions of the importance of specific biosecurity practices and the likelihood of future AI outbreaks and communication networks and information sources. In relation to the questions on perceptions, farmers were asked to rate the importance of biosecurity practices using a scale from one to five; one being ‘not at all important’ and five being ‘extremely important’. In addition, farmers were asked to give a personal biosecurity rating of their farm. The rating was from one to five; one being ‘you do not believe you follow biosecurity measures at all’ and five being ‘you believe you accomplish all biosecurity measures thoroughly’. Farmer opinion was also obtained on the likeliness (‘not at all likely’, ‘unlikely’, ‘likely’, ‘very likely’ and ‘unsure’) that an AI outbreak will occur on their farm and the likeliness that an AI outbreak will occur in Australia within the next year. An open question was asked on the level of santisation performed after the depopulation of sheds. Answers which described a high level of cleaning performed between each batch were categorized as ‘thorough sanitisation’. All other answers were categorized into ‘partial sanitisation’.

After conducting the interviews, researchers recorded visual observations of the farm including shed and range design, waterbody locations and biosecurity practices seen during farm walks. The experimental procedures used for this survey were approved by the Human Ethics Committee of the University of Sydney, Australia and all results obtained were kept confidential. Written consent for farmer participation in the survey was obtained by the researchers (ethics reference number: 2015/252).

### Data analysis

Data from the questionnaires and observation records were entered in Microsoft Access (Microsoft, PC/Windows 7, 2010, Redmond, WA, USA) after each farm visit and checked for data entry errors. The statistical program JMP® was used (2012 SAS Institute Inc., Cary, USA) for all statistical analyses of the data. One-way analyses of variance were used to determine statistically significant differences in the distances between farms, sheds and sheds and waterbodies amongst the different farm types, the number of waterbodies on the property, turnaround times, personal farm biosecurity ratings and percentage of birds affected by unusual signs amongst the different farm types. The Pearson’s chi-square test was used to determine statistically significant differences amongst the different farm types for the categorical answers, including water sources, depopulation and sanitisation methods, vehicle and equipment disinfection, and personnel biosecurity practices. A bivariate analysis was performed to determine any statistical association between the number of chickens on the farm and the personal biosecurity rating overall and per farm type. In all statistical analyses, a P-value of <0.05 was considered significant.

Univariable logistic regression analyses were used to estimate the association between reported compliance of implementation of biosecurity practices and farmer perceived importance of these practices, and to determine the relationship between the presence of waterfowl around feed storage areas and on the range and the minimum distance between sheds and a waterbody. ‘Unsure’ answers were omitted from all analyses.

## Results

### Brief description of farms

The farms surveyed were all commercially operating farms. The median number of chickens was greatest on meat chicken farms (140,600 and 88,000 chickens for free range and barn meat chicken farms respectively). This was then followed by cage layer, free range layer and barn layer farms (40,000, 32,000 and 17,500 chickens respectively). The median number of sheds was four and five for barn meat chicken and free range meat chicken farms respectively. The median number of sheds for layer farms was two for both cage and barn layer farms and three for free range layer farms.

### Biosecurity

Across all farm types, all but one free range layer farm had some understanding of and could adequately define the term ‘biosecurity’. Biosecurity manuals were present on all meat chicken farms surveyed, and the majority of barn layer farms (89%) and free range layer farms (88%) and half (50%) of cage layer farms reported having a biosecurity manual on the farm.

#### Waterbodies and distances between sheds and other farms

The overall median number of dams across all farm types was one. The overall median distance between a study farm and the closest commercial farm in this survey was one kilometre (km); ranging from 0.8 km for barn meat chicken and cage layer farms to 1.5 km for free range layer farms. Cage layer farms had the shortest median distance between sheds (7.75 metres (m)) and between sheds and waterbodies (30 m) compared to the other farm types. The distances between sheds was significantly different between farm types (P<0.05) (Table 1).

**Table 1:** Biosecurity information related to farm layout, water sources, shed/vehicle/equipment sanitation and personnel practices on commercial layer and meat chicken farms in the Sydney basin and South East Queensland during 2015–2016.

Farm feature / management practice	Farm type					P-value
	Barn meat chicken (n = 15)	Free range meat chicken (n = 15)	Cage layer (n = 9)	Barn layer (n = 9)	Free range layer (n = 25)	
Median distance (range)						
Between farms (km)*	0.8 (0.1–3)	1 (0.1–5)	0.8 (0.1–15)	1.2 (0.1–25)	1.5 (0.1–25)	0.59
Between sheds (m)	9 (6–18)	15 (9–29.5)	7.8 (0–13.5)	10 (0–20)	13 (0–30)	<0.05
From shed to waterbody (m)	62 (2.5–350)	60 (20–850)	30 (5.5–103)	121 (32–5000)	100 (25–370)	0.07
Water						
Median number of dams (range)	1 (0–5)	2 (0–5)	1 (0–2)	1 (0–3)	1 (0–4)	0.40
Drinking water source (%)						<0.05
Town water	60 (n = 9)	40 (n = 6)	100 (n = 9)	67 (n = 6)	68 (n = 17)	
Farm dam	7 (n = 1)	0	0	0	16 (n = 4)	
Bore water	33 (n = 5)	47 (n = 7)	0	11 (n = 1)	8 (n = 2)	
River/ creek	0	13 (n = 2)	0	22 (n = 2)	8 (n = 2)	
Drinking water not treated	13 (n = 2)	0	0	0	4 (n = 1)	
Secondary water source used (%)						0.33
Farm dam	0	13 (n = 2)	22 (n = 2)	0	4 (n = 1)	
River/ creek	0	0	0	11 (n = 1)	4 (n = 1)	
Bore water	0	0	0	0	4 (n = 1)	
Secondary water source not treated	N/A	50 (n = 1)	100 (n = 2)	100 (n = 1)	100 (n = 3)	
Depopulation, shed sanitisation and turnaround times (%)						
Depopulation of birds occurs in one day (%)	20 (n = 3)	0	22 (n = 2)	89 (n = 8)	88 (n = 22)	<0.05
Thorough sanitisation (%)	93 (n = 14)	100 (n = 15)	33 (n = 3)	89 (n = 8)	88 (n = 22)	<0.05
Average turnaround time in days (range)	10.8 (7–14)	10.6 (7–13)	10.5 (0–25)^c^	23.1 (0–35)	22.9 (0–35)	<0.05
Vehicle and equipment disinfection (%)						
Disinfection of vehicles between farms	85 (n = 11/13) ^a^	43 (n = 6/14) ^a^	63 (n = 5)	67 (n = 6)	48 (n = 12)	0.24
Sharing equipment between sheds	73	60	78	78	92	0.21
Disinfection of equipment between sheds	45 (n = 5/11)^b^	0 (n = 0/9)^b^	0 (n = 0/7)^b^	14 (n = 1/7)^b^	9 (n = 2/23)^b^	<0.05
Personnel biosecurity practices (%)						
Foot baths used	93 (n = 14)	93 (n = 14)	25 (n = 2)	67 (n = 6)	76 (n = 19)	<0.05
Hand washing/ sanitation facilities	100 (n = 15)	100 (n = 15)	89 (n = 8)	89 (n = 8)	96 (n = 24)	0.40
Visitor recording book	93 (n = 14)	100 (n = 15)	25 (n = 2)	56 (n = 5)	80 (n = 20)	<0.05
Change of clothes between farms	93 (n = 14)	100 (n = 15)	75 (n = 6)	67 (n = 6)	58 (n = 14/24)^a^	0.06
Farms provide clothing to workers/visitors	62 (n = 8/14) ^a^	93 (n = 14/15) ^a^	29 (n = 2/8) ^a^	33 (n = 2/6) ^a^	24 (n = 5/21) ^a^	<0.05
Restricted contact between farms	77 (n = 11/14) ^a^	100 (n = 14/15) ^a^	89 (n = 8)	100 (n = 8/8) ^a^	67 (n = 16/24) ^a^	0.13

* Between farms refers to distance between the farm interviewed to another commercial poultry farm

a There is missing data for some questions on the biosecurity questionnaire. Although researchers were present to discuss and explain the questionnaire to the farmers; some farmers chose to leave some questions blank even after discussion.

b Farmers were asked if equipment was dedicated per shed or there was sharing of equipment between sheds. These percentages are those that share equipment between sheds only.

c This average turnaround time includes the length of time cages are empty in multi-aged sheds i.e. birds are still present inside sheds in other cages.

#### Water source and treatment

The source of the drinking water for chickens was town water for the majority of all farm types; the percentages for each farm type are shown in Table 1. Bore water was the second most common drinking water source and two barn meat chicken farms and one free range layer farm did not treat the drinking water which was sourced from bore water. All other farms treated their drinking water. Most farms used the drinking water source for all other features on the farm that required water such as foggers, irrigation, sprinklers and cooling pads. However, the use of different water sources for features on the farm other than drinking water, known as secondary water sources, was found on all farm types except barn meat chicken farms. All farms that used secondary water sources did not treat the water except for one free range meat chicken farm (Table 1). For farms that performed water treatment, chlorination was the most popular method (81%). The number of farms that perform irrigation and use the different environmental control methods including foggers, sprinklers and cooling pads can be found in Scott et al. 2017.

#### Depopulation, shed sanitisation and turnaround times

The percentage of farms that depopulated entire sheds in one day differed significantly by farm type (P<0.05) and occurred most commonly in barn layer farms (89%) and free range layer farms (88%) compared to other farm types (Table 1). Most cage layer farms (78%) had multi-aged sheds and depopulated birds of a single tier or row simultaneously; only two cage layer farms (22%) depopulated entire sheds in one day. Thinning out is most commonly practiced on meat chicken farms where birds are depopulated over more than two weeks; few barn meat chicken farms (20%) and no free range meat chicken farms reportedly perform depopulation of entire sheds in one day. Performing thorough sanitisation of sheds after depopulation of entire sheds was also significantly different amongst the farm types (P<0.05), with most barn and free range meat chicken farms and layer farms performing this practice compared to only a third of cage layer farms (Table 1). In relation to turnaround times, described as the time period from when sheds are depopulated of birds to when new birds arrive, meat chicken farms had lower (P<0.05) average turnaround times (approximately 10 days) than barn and free range layer farms (approximately 23 days). However, there was one barn and one free range layer farm with no turnaround times, introducing new birds the same day that the previous batch was removed. For cage layer farms, the average turnaround time of empty cages i.e. including multi-aged sheds where birds are still present inside the sheds was 10.5 days. However, the average turnaround time was 22.8 days when only considering the two cage farms that depopulate the entire shed in one day (Table 1).

#### Vehicle and equipment disinfection

Types of vehicle disinfection described included wheel washes, wheel dips and whole vehicle washes. Information on whether or not vehicle disinfection was performed before or after visiting the farm was captured. The majority of barn meat chicken farms (85%) reported that disinfection of vehicles occurred before or after visiting their farm compared to a lower proportion of the other farm types, as described in Table 1. Sharing equipment between sheds was common in all farm types, with the highest proportion of farms conducting this practice being reported among barn meat chicken farms (92%), with the lowest being among free range chicken meat farms (60%).

Farms that did not share equipment between sheds, reportedly had dedicated equipment per shed. Disinfection of shared equipment was reportedly low across all farm types; however, it differed among farm types (P< 0.05). While almost half of barn meat chicken farms would disinfect shared equipment, a very low proportion of barn and free range layer and no free range meat chicken or cage layer farms, would conduct this practice (Table 1).

#### Personnel biosecurity

Footbath presence on farms varied significantly from 93% on both meat chicken farm types to only 25% of cage layer farms (P<0.05). Hand washing/ sanitation facilities were present on 100% of both meat chicken farm types and 96%, 89% and 88% of free range layer, barn layer and cage layer farms respectively. Visitor recording was present in 100% and 93% of free range and barn meat chicken farm types respectively, followed by free range layer (80%), barn layer (56%) and then in only 25% of cage layer farms (P<0.05). Changing clothes between farms when visiting farms within the same company occurred more commonly on meat chicken farms (93% and 100% for barn and free range meat chicken farms respectively) than layer farms (75%, 67% and 58% in cage, barn and free range layer farms respectively) (P<0.05). Protective clothing was provided to workers and visitors on 93 free range meat chicken farms as compared to 24% (n = 5/21) free range layer farms. The minimum waiting period between visiting poultry farms owned by different poultry companies was at least 48 hours across all farm types. This restricted contact between farms applied to all visitors including trained depopulation, shed sanitisation and vaccination crews and was reportedly enforced in 100% of free range meat chicken (n = 14/15) and barn layer (n = 8/8) farms (Table 1).

### Biosecurity ratings

#### Farm perceived importance of biosecurity practices

**Fig 1:**
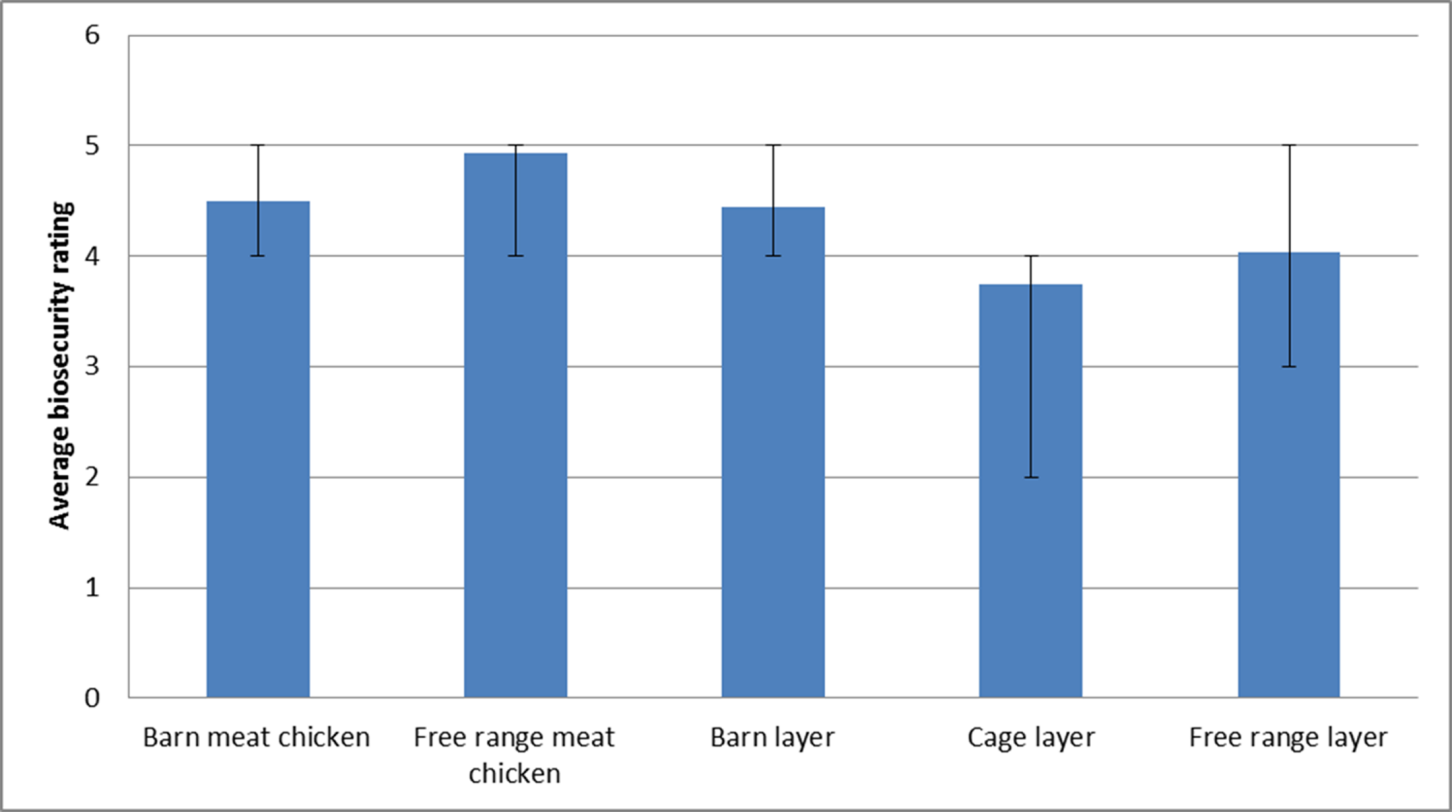
Overall perceived importance of biosecurity practices per farm type (most biosecurity practices rated as ‘very important’ including those not depicted).

Across the farm types, most biosecurity practices were rated on average as ‘very important' by farmers. Some practices gained an average rating of ‘extremely important’ but only by meat chicken farms. Of note was the relatively low rating of importance for disinfection of equipment between sheds by barn meat chicken, cage layer and free range layer farm types. Visitor recording and turnaround times in sheds were also rated relatively low by cage layer farms (Fig 1).

#### Personal farm biosecurity ratings and future risk of AI outbreaks

**Fig 2:**
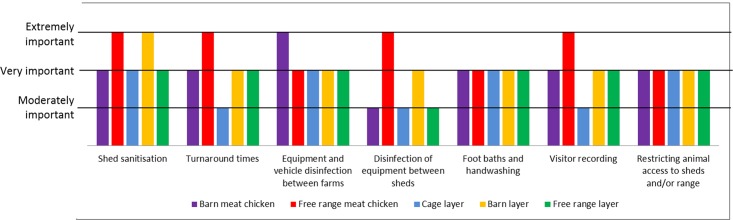
Average farmer rating of farm biosecurity compliance per farm type (P<0.05).

Perceived level of implementation of on-farm biosecurity differed among the farm types (P<0.05). Cage layer farms had the lowest average personal farm biosecurity rating (3.75, 95% CI 3.16–4.34), with free range meat chicken farms having the highest average rating (4.93, 95% CI 4.77–5.00) (Fig 2).A bivariate analysis revealed no statistically significant association between the number of chickens on the farm and the personal farm biosecurity compliance rating and this was still the case when performed by farm type.

Thirty nine percent of farmers considered it “unlikely” that an AI outbreak would occur on their own farm, while 33% were unsure. Forty percent of farmers across all farm types believed it was ‘likely’ that an AI outbreak would occur on another farm in Australia within the next year, while 21% believed it was ‘unlikely’.

### Wild and domestic animal presence

Table 2 describes farmer reporting of wild and domestic animal presence on farm. Most farms reported sighting insects and/or spiders inside sheds and similarly, small mammals, such as rats and mice in sheds were also reported across all farm types, being least common in barn meat chicken farms (67%). Large mammals, mainly foxes, were also reported across all farm types, most often on the outskirts of the shed, rarely accessing the inside. Rodent control was common across all farm types, being performed on 96% of farms.

**Table 2:** Reported wild animal presence in sheds, feed storage or range areas, domestic animal presence on farm, and dog/cat access to sheds or range areas on commercial layer and meat chicken farms in the Sydney basin and South East Queensland during 2015–2016.

Animal type reported on farm & dog/cat access to areas on farm	Farm type (%)					P-value
	Barn meat chicken (n = 15)	Free range meat chicken (n = 15)	Cage layer (n = 9)	Barn layer (n = 9)	Free range layer (n = 25)	
Wild animals						
Insects in sheds	93 (n = 14)	87 (n = 13)	100 (n = 9)	100 (n = 9)	100 (n = 25)	0.26
Small mammals (e.g. rats, mice) in sheds	67 (n = 10)	80 (n = 12)	89 (n = 8)	89 (n = 9)	76 (n = 19)	0.65
Large mammals (e.g. foxes) on farm	27 (n = 4)	40 (n = 6)	67 (n = 6)	44 (n = 4)	36 (n = 9)	0.40
Wild birds in sheds	47 (n = 7)	73 (n = 11)	56 (n = 5)	56 (n = 5)	52 (n = 13)	0.64
Wild birds on range		87 (n = 13)			88 (n = 22)	0.90
Wild birds on waterbodies	87 (n = 13)	93 (n = 14)	100 (n = 9)	78 (n = 7)	96 (n = 24)	0.37
Wild birds on feed storage areas	73 (n = 11)	80 (n = 12)	89 (n = 8)	89 (n = 8)	72 (n = 18)	0.74
Domestic animals present on farm						
Dogs/Cats	67 (n = 10)	80 (n = 12)	89 (n = 8)	56 (n = 5)	68 (n = 17)	0.51
Ruminants	67 (n = 10)	60 (n = 6)	56 (n = 5)	11 (n = 1)	32 (n = 8)	<0.05
Horses	7 (n = 1)	7 (n = 1)	11 (n = 1)	0	28 (n = 7)	0.13
Pigs	0	0	0	0	4 (n = 1)	0.75
Dog/cat access to chicken facilities						
Access to sheds	0	7 (n = 1)	67 (n = 6)	11 (n = 1)	8 (n = 2)	<0.05
Access to range		7 (n = 1)			44 (n = 11)	<0.05

Wild birds inside sheds were most commonly reported in free range meat chicken farms (73%) compared to approximately half of farms for the other farm types. All wild birds reported to be seen inside sheds were small bird types such as sparrows and finches. Wild birds on the range were also commonly reported for both layer and meat chicken free range farms, with a wide range of species being reported, including ducks, plovers, crows and pigeons. Waterfowl were reported as the most common wild bird species appearing on nearby waterbodies. Species most commonly reported around feed storage areas were pigeons or mynahs. Fifty-eight percent of farmers across all farm types reported that wild birds commonly visited after feed spills. For the other 42%, this was prevented due to prompt cleaning of the feed spill. Wild bird proofing of sheds was common across farm types with 86% of farms overall reporting this to be done.

Dogs and cats were kept by the majority of farms across all farm types (71%). This was followed by ruminants (45%), horses (14%), other animals (5%) and pigs (1%). The pigs were reported on a free range layer farm. Dog and cat access to sheds and the range were both significantly different amongst the farm types (P<0.05), with access to the sheds in the majority (67%) of cage layer farms and access to the range in 44% of free range layer farms (Table 2).

### Disease

#### Detection and reporting on unusual signs in a flock

Farmers were asked to describe the most significant unusual signs in a flock from their perspective. This information is presented in Table 3. The most commonly reported signs were respiratory signs for barn layer (56%) and free range meat chicken farms (40%), lethargy for barn meat chicken farms (73%), and mortalities for free range layer farms (52%). For cage layer farms, lethargy and reduction in egg production were equally the most common unusual signs (56%).

**Table 3:** The five most common unusual signs^a^ perceived by farmers as most significant and the number and percentage of chickens in a shed affected by unusual signs that would prompt the farmer to contact someone on commercial layer and meat chicken farms in the Sydney basin and South East Queensland during 2015–2016.

Unusual sign / Bird number	Farm type (%)				
	Barn meat chicken (n = 15)	Free range meat chicken (n = 15)	Cage layer (n = 9)	Barn layer (n = 9)	Free range layer (n = 25)
Unusual signs					
Lethargy	73 (n = 11)	33 (n = 5)	56 (n = 5)	44 (n = 4)	44 (n = 11)
Respiratory	53 (n = 8)	40 (n = 6)	56 (n = 5)	56 (n = 5)	36 (n = 9)
Ocular signs^b^	47 (n = 7)	33 (n = 5)	44 (n = 4)	44 (n = 4)	-
Abnormal gait	33 (n = 5)	-	-	-	-
Decreased feed consumption	27 (n = 4)	33 (n = 5)	33 (n = 3)	33 (n = 3)	-
Decreased growth rate	-	33 (n = 3)	-	-	40 (n = 10)
Mortalities	-	-	33 (n = 3)	44 (n = 4)	52 (n = 13)
Egg production drop	-	-		33 (n = 3)	48 (n = 12)
Number of birds affected to prompt action					
1 to 10 birds	7 (n = 1)	0	56 (n = 5)	44 (n = 4)	48 (n = 12)
10 to 50 birds	46.5 (n = 7)	40 (n = 6)	33 (n = 3)	44 (n = 4)	44 (n = 11)
>50 birds	46.5 (n = 7)	60 (n = 9)	11 (n = 1)	11 (n = 1)	8 (n = 2)
Median percentage of birds in the shed affected by unusual signs to prompt action	0.21 (0.01–10)	0.29 (0.06–5)	0.21 (0.0025–5)	0.09 (0.01–12.5)	0.06 (0.01–5)

a Only the five most common unusual signs per farm type are recorded. In the case of barn layer farms, the fifth sign was tied between two clinical signs and therefore six entries are shown for this farm type.

b Ocular signs included anything abnormal with the eyes, including discharge, squinting and redness.

When asked how many birds in a shed affected by unusual signs would prompt the farmer to contact someone, most layer farms reported taking action if up to 10 birds were affected while the vast majority of meat chicken farms did so when 50 or more birds in a shed were affected. Table 3 presents the median and range percentage of birds in a shed affected by unusual signs that would prompt the farmer to contact someone. The percentage for barn and free range layer farms (0.09% and 0.06% respectively) is less compared to barn and free range meat chicken farms (0.21% and 0.29% respectively). The median percentage for cage layer farms is the same as barn meat chicken farms (0.21%).

Across all farm types, a service person was the most commonly reported person that the farmer would contact (44%), followed by the farm manager/owner (22%), a veterinarian (20%) and a company representative (15%). Answers varied depending on the ownership situation of the farm. Most farms surveyed were company-owned and had service people that were the first contact for farmers, followed by the company veterinarian. For small, individual farms, sales representatives who farmers bought chickens from, farm managers/owners or independent veterinarians would be contacted directly.

#### Perceptions on AI and other diseases

Most farmers were concerned about AI and Newcastle Disease (77% and 66%, respectively. across all farm types). Infectious Laryngotracheitis (ILT) was another disease of concern, however, the level of concern differed according to farm type. This disease was listed as concern for most barn and free range meat chicken farmers (87% of farmers for both farm types), 67% of cage layer farms and only 32% of free range layer farms. A proportion of free range layer farmers (28%) were also worried about spotty liver disease. Scott et al. 2017 provides information on vaccines used on these farms.

The most popular answer to describe signs in chickens that the farmers would associate with AI infection was mortalities for free range meat chicken and cage layer farms (53% and 33% respectively) and respiratory signs for barn meat chicken, barn layer and free range layer farms (60%, 56% and 44% respectively).

### Relationships between wild animals, biosecurity practices, perceptions, and farm design

#### Relationships between reported compliance and farmer perception of biosecurity practices

Results of univariable logistic regression analysis to determine the association between reported compliance with a biosecurity practice on farm and farmer-perceived importance of the biosecurity practice are presented in Table 4. The model was unstable when performed for the biosecurity practice of hand washing, and therefore the results were omitted. There were significant statistical associations between reported compliance and the perceived importance of all biosecurity practices listed except for the practice of disinfecting equipment between sheds (P = 0.71). Except for rodent control and wild bird proofing sheds, all significant associations were positive, meaning that when the practice was present, the importance rating was higher.

**Table 4:** Association between reported compliance with a biosecurity practice on farm (Yes/No) and farmer-perceived importance of the biosecurity practice (rated 1 to 5) on 73 commercial layer and meat chicken farms in the Sydney basin and South East Queensland during 2015–2016.

Biosecurity practice	β	SE (β)	Unit odds ratio (95% CI)	P-value
Restricting animal access to sheds and/or range	2.38	0.91	10.81 (2.68–73.31)	<0.05
Footbaths	0.93	0.30	2.54 (1.47–4.83)	<0.05
Visitor recording	0.74	0.23	2.1 (1.35–3.42)	<0.05
Change clothes between farms within same company	1.12	0.47	3.08 (1.29–8.41)	<0.05
Provide clothing to workers/ visitors	1.12	0.35	3.06 (1.65–6.61)	<0.05
Restricted contact between farms	3.08	0.85	21.75 (5.59–171.54)	<0.05
Disinfection vehicles between farms	1.85	0.44	6.34 (2.96–16.97)	<0.05
Disinfection equipment between sheds	-0.14	0.37	0.87 (0.38–1.73)	0.71
Rodent control	-2.18	1.01	0.11 (0.005–0.58)	<0.05
Wild bird proofing sheds	-2.40	0.70	0.09 (0.01–0.27)	<0.05

The significant negative association between rodent control practice and perceived importance of rodent control (P<0.05, OR 0.11 (Table 4)) was reinforced by the non-significant association between the presence of rodents in sheds and perceived importance of control of rodents in sheds (P = 0.18) (Table 5). For wild birds, there was a significant negative association between the presence of wild birds in sheds and perceived importance of wild bird control (P<0.05, OR 0.5), meaning when farmers reported wild bird presence in sheds, they were less likely to award a higher importance rating for wild bird control (Table 5).

**Table 5:** Association between presence of wild animals in sheds (Yes/No) and farmer-perceived importance of wild animal control in sheds (rated 1 to 5) on 73 commercial layer and meat chicken farms in the Sydney basin and South-East Queensland during 2015–2016.

Wild animal type	β	SE (β)	Unit odds ratio (95% CI)	P-value
Wild birds	-0.69	0.28	0.5 (0.27–0.83)	<0.05
Rodents	0.41	0.31	1.51 (0.81–2.8)	0.18

#### Relationships between waterfowl presence and farm design factor

Univariable logistic regression analyses between reported presence of waterfowl around feed storage areas (Yes/No) and on the range (Yes/No) and the minimum distance between sheds and a waterbody revealed P-values of 0.55 and 0.06 respectively. This indicates there is no statistically significant evidence that the minimum distance between sheds and a waterbody influences the reported presence of waterfowl around feed storage areas or on the range.

## Discussion

This survey expands knowledge of the biosecurity practices performed on Australian commercial chicken meat and layer farms, and farmer-perceived importance of these practices. Identification of the biosecurity practices with lower compliance enables emphasis on these practices in educational efforts, such as biosecurity workshops and information sessions performed by Australian chicken egg and meat industries, and lower compliance for certain farm types also enables specific sectors of the industry to be targeted for participation in such programs. For some farmer participants in this survey, simply completing the biosecurity questionnaire led to realisation of gaps in their biosecurity practices.

The sample of farms used in this survey is likely representative of the commercial chicken egg and meat farms across Australia. The survey was performed mainly in NSW in the Sydney basin region and a large range of farm sizes and companies were visited. NSW is the leading state in both the number of farms and volume of product produced for meat chicken and egg farms in Australia [21–23]. However, care should be taken when extrapolating results from this survey to areas of Australia that have experienced the recent establishment of new farms as farm layout and structure of farm buildings heavily influences biosecurity practices conducted on them [4, 10].

Non-compliance with biosecurity practices has been previously related to inadequate training and education of farmers and limited communication between farmers and technical service providers [10]. We found overall a high level of biosecurity compliance among the meat chicken farms, likely due to the vertical integration of this sector. The high level of private ownership amongst the layer farms is considered to contribute to greater variation in the level of biosecurity as there is no mandatory governing body to enforce adoption and compliance of biosecurity practices [4, 10]. Egg Corp Assured is a national quality assurance program developed to help commercial egg producers improve issues including food safety and biosecurity. However, while progressive, it does not cover all producers [10]. Cage layer farms tended to rate lowest in the level of biosecurity amongst the farm types. The results from the farmer assessment of biosecurity compliance rating of their farm also reflect this; cage layer farmers gave their farms the lowest average rating compared to the other farm types. This demonstrates some degree of awareness of farm biosecurity amongst the cage farmers and an acknowledgment of lower compliance than best practice. Most cage layer farms visited in this survey were old, family-run farms that had been passed onto the next generation and this is a particular feature of farms located in the Sydney basin region in general [24]. This contrasts with barn and free range layer farms which are relatively new due to the recent expansion of cage-free egg production [13]. It is probable that the structural features of older farms to some extent limit compliance with current best practice, and likely that farmers with more recently established farms seek technical services and support more frequently than farmers with long established farms. However, the age of the farms as well as the level of biosecurity training received by farmers and other staff members was not captured in this survey.

There was at least one dam, on average, on every farm type. Dams are generally used on farms as water reservoirs or catchments [10]. Five farms used them as the source of drinking water for chickens in this survey. The majority of farms across all farm types use town water which is treated with chlorine and occasionally ammonia in the Sydney basin region [25]. As the presence of dams attracts wild birds, particularly waterfowl, on to a farm, their presence is a potential biosecurity risk due to the potential for introduction of pathogens to chickens on the farm. Potential pathways include contact of free range chickens with waterfowl on the range, or the consumption of contaminated drinking water from the dams. For the latter, it is especially prudent that farmers treat water sourced from dams to reduce this potential. A national water biosecurity manual was published in 2009 to assist Australian poultry producers with performing appropriate water biosecurity practices [26]. Aligning with the recommendations of this manual, this survey found a high level of water treatment across all farm types.

The majority of cage layer farms had multi-age sheds (78%) where each row or tier is a different age group of chickens in order to maintain the flow of egg production [10]. This generally means there are always chickens in sheds as new ones replace the old, and thus sheds are rarely empty. This explains why few cage layer farms (33%) perform thorough sanitisation of sheds between batches. From a disease view-point, this allows persistence and circulation of pathogens. Of note is AI, where once LPAI is introduced into a flock, it is generally acknowledged that persistent circulation of the virus in a population of poultry leads to a greater risk of mutation to HPAI [27]. AI was reported as the most worrying poultry disease to farmers but this is likely due to the survey being AI focused, as farmers were told about the research project. ILT was also a common concern amongst farmers; likely due to the ILT outbreaks occurring across the Sydney basin region at the time this survey was conducted [28]. Farmers that listed ND as a concern tended to be those that had experienced the ND outbreaks which occurred between 1998 and 2002 in NSW and Victoria [4].

The presence of dogs, cats and ruminants was common across all farm types. The majority (67%) of cage layer farmers allowed dog and cat access inside sheds on the chicken farm, and for cats this was reported to contribute to rodent control. However cats can potentially serve as carriers of virulent forms of *Pasteurella multocida* and *Toxoplasma gondii* infection for chickens [29, 30]. Forty-four percent of free range layer farms also allowed dog access to the range, mainly as a form of protection for the chickens from potential predators, especially foxes. Of the 12 farms that allowed dog access to the range in this survey, 16% (n = 2) had the Maremma Italian guard dog; its use is relatively recent in Australia. There is evidence for this breed’s effectivity in protecting a range of livestock species from several types of predators [31]. One free range layer farm had pigs on the farm. Keeping pigs and poultry in the same premises is a major biosecurity concern. The national farm biosecurity manual for the Australian poultry industry states that this should not occur and that farm staff must not come into contact with pigs or other avian species [1]. This is because of the potential of salmonellosis in poultry flocks from pigs or vice versa [32]. In addition, pigs are susceptible to infection of both avian and human influenza viruses; thereby they can act as “mixing vessels” where novel re-assortment of influenza viruses can occur. There is fear this can lead to influenza viruses transmitted from pigs that are more readily spread between mammals [33].

It was conjectured by the authors that larger farms would be more likely to seek technical support and services and therefore have improved biosecurity compared to smaller farms. This is because smaller farms usually develop first as hobby farms which gradually increase in size; hobby farmers usually have limited knowledge and experience compared to farm staff on large commercial chicken farms [10]. However, the results of this survey revealed there was no significant statistical association between the number of chickens on farm and the farmer biosecurity compliance rating of their farm. There may be some degree of modesty in the results, where well-educated farmers do not rate their farm highly as they believe there is room for improvement on the current farm.

The finding that reported compliance with a biosecurity practice on farm and farmer-perceived importance of the biosecurity practice are positively associated can guide decisions on biosecurity practices for inclusion in educational efforts for farmers. The results revealed that for most biosecurity practices, when the practice was performed, the farmer rating of importance was higher. Thus, encouraging biosecurity practices to be performed on farm can improve farmer-perceived importance of certain practices, leading to long term performance. In contrast, there was a significant negative association between rodent control and its farmer-perceived importance. There was also no significant statistical association between the perceived importance of rodent control in sheds and the reported presence of rodents. Vermin control was rated on average as very or extremely important across the farm types and there was a high percentage of rodent control performance across all farm types; 96% of farms overall. Farmer-perceived importance of rodent control may be clouded by failure to reduce rodent numbers despite implementing rodent control, due to the ideal conditions for rodent presence and breeding on farms in general and the possible resistance to rodent baits [34].

There was a significant negative association between compliance of wild bird proofing sheds and farmer-perceived importance of wild bird proofing sheds. There was also a significant negative association between wild bird presence in sheds and farmer-perceived importance of wild bird proofing sheds. This means that when farmers either performed wild bird proofing of sheds or reported wild birds in sheds, their perceived importance of wild bird control was lower. Reasons for these associations may be due to general unawareness of the significance of wild bird presence inside sheds in terms of pathogen transfer. These results touch on the complex topic of factors that influence biosecurity compliance on farms by farmers; such factors include the perceived threat of disease, ease and benefits of performing the practice, and personality traits [35]. Studies in Canada on poultry farms revealed that the personality traits of complexity, responsibility, work experience and education were positively correlated with biosecurity compliance [36]. A low perceived threat of wild bird presence inside sheds, due to a lack of education of the pathogens potentially transferable from wild birds, is likely to play a role in contributing to the low farmer-perceived importance of wild bird proofing sheds in this survey. Improving farmer compliance of biosecurity practices that reduce wild animal visits to poultry farms is important in reducing the potential of infectious disease introduction and spread to poultry [10].

Although the statistical significance of the association between the minimum distance from a shed and a waterbody and waterfowl presence on the range in this survey was limited (P = 0.06), this tendency suggests that the distance between sheds and waterbodies may influence the presence of waterfowl on range areas. Direct contact between chickens and waterfowl on range areas can lead to the transmission of pathogens [37]. Pathogen transmission via aerial dispersion, although rare, is also possible such as from LPAI-infected waterfowl [7, 38]. If the distance between sheds and waterbodies does indeed influence waterfowl presence on range areas, then the location of waterbodies can be changed by farmers to be located further away from range areas. The goal in changing the location of waterbodies being to reduce waterfowl presence and the potential of pathogen transmission via aerial dispersion. Waterfowl movements and presence are also known to be affected by other factors such as rainfall, feed, and open spaces on-farm; and this can be explored through further research [39].

The purpose of conducting on-farm visits and interviews rather than telephone interviews was to eliminate answers which the farmer may wish to report but did not hold true. Many biosecurity practices could be noted as present or absent during the on-farm visits by the researchers, such as footbaths, hand washing facilities, visitor recording systems and domestic animals on the farms. However some practices, which are largely dynamic or which occurred only at specified times, could not be observed, such as thorough shed sanitisation between batches and vehicle/equipment disinfection between sheds and farms. In these instances true answers were dependent on accurate farmer reporting.

In summary, this survey provides further knowledge on the level of adoption of biosecurity practices and farmer-perceived importance of these across commercial meat and layer chicken farms in Australia. This knowledge helps identify practices and farm types in which biosecurity needs most improvement. A high level of biosecurity was found in meat chicken farm types compared to layer farm types, and cage layer farms have the largest room for improvement. Educational efforts such as workshops or information sessions can be conducted to improve the level of adoption and compliance of biosecurity practices. Improving the level of biosecurity practices will reduce the potential for introduction and spread of infectious diseases in Australian commercial chicken farms.
